# Integrator complex subunit 6 (INTS6) inhibits hepatocellular carcinoma growth by Wnt pathway and serve as a prognostic marker

**DOI:** 10.1186/s12885-017-3628-3

**Published:** 2017-09-12

**Authors:** Ka Yin Lui, Hui Zhao, Chunhui Qiu, Chuo Li, Zhigang Zhang, Haoran Peng, Rongdang Fu, Hu-an Chen, Min-qiang Lu

**Affiliations:** 1 Department of Hepatobiliary Surgery, Guangzhou First People’s Hospital, Guangzhou, 510180 China; 2 Department of Critical Care Medicine, the First Affiliated Hospital of Sun Yat-sen University, Guangzhou, 510080 China; 3 Department of Hepatic Surgery, the Third Affiliated Hospital of Sun Yat-sen University, Guangzhou, 510630 China; 4 Obstetric Laboratory, the Third Affiliated Hospital of Sun Yat-sen University, Guangzhou, 510630 China; 5 Transitional Year, Gwinnentt Medical Center, Lawrenceville, GA USA; 6 Department of Pathology, the Third Affiliated Hospital of Sun Yat-sen University, Guangzhou, 510630 China

**Keywords:** INTS6, Hepatocellular carcinoma, Prognosis, Wnt/β-catenin

## Abstract

**Background:**

Integrator complex subunit 6 (INTS6) was found to play a tumour suppressing role in certain types of solid tumours. In this study, we wanted to determine the expression level of INTS6 in hepatocellular carcinoma (HCC) and evaluate its clinical characteristics and mechanisms in HCC patients (Lui and Lu, European Journal of Cancer, 51:S94, 2015).

**Methods:**

First, we used a microarray analysis to explore the mRNA expression levels in HCC and paired normal liver tissues; second, we used qRT-PCR to measure the INTS6 mRNA levels in a cohort of 50 HCC tissues and adjacent normal liver tissues; third, we used Western blot analyses to detect the INTS6 protein levels in 20 paired HCC and normal liver tissues; fourth, we used immunohistochemistry to determine the INTS6 expression levels in 70 archived paraffin-embedded HCC samples. Finally, we investigated the suppressive function of INTS6 in the Wnt pathway.

**Results:**

Herein, according to the microarray data analysis, the expression levels of INTS6 were dramatically down-regulated in HCC tissues vs. those in normal liver tissues (*p*<0.05). qRT-PCR and Western blot analyses showed that the INTS6 mRNA and protein expression was significantly down-regulated in tumour tissues compared to the adjacent normal liver tissues (*p*<0.05). Immunohistochemical assays revealed that decreased INTS6 expression was present in 62.9% (44/70) of HCC patients. Correlation analyses showed that INTS6 expression was significantly correlated with serum alpha-fetoprotein levels (AFP, *p* =0.004), pathology grade (*p* =0.005), and tumour recurrence (*p* =0.04). Kaplan-Meier analysis revealed that patients with low INTS6 expression levels had shorter overall and disease-free survival rates than patients with high INTS6 expression levels (*p* =0.001 and *p* =0.001). Multivariate regression analysis indicated that INTS6 was an independent predictor of overall survival and disease-free survival rates. Mechanistically, INTS6 increased WIF-1 expression and then inhibited the Wnt/β-catenin signalling pathway.

**Conclusion:**

The results of our study show that down-regulated INTS6 expression is associated with a poorer prognosis in HCC patients. This newly identified INTS6/WIF-1 axis indicates the molecular mechanism of HCC and may represent a therapeutic target in HCC patients.

**Electronic supplementary material:**

The online version of this article (10.1186/s12885-017-3628-3) contains supplementary material, which is available to authorized users.

## Background

Hepatocellular carcinoma (HCC) is one of the most common cancers in the world and has characteristics of high mobility, high recurrence rates, and poor prognosis [1]. Approximately 110,000 people die of HCC each year in China [2]. This mortality rate accounts for 45% of the total deaths from HCC in the world. Potentially curative therapies for HCC include surgical resection and liver transplantation [3]. In recent years, tremendous progress has been made towards understanding the causes of HCC, such as hepatitis B virus or hepatitis C virus infection, alcohol consumption, and water contamination [4, 5]. The discovery of some significant causes of HCC makes this disease somewhat preventable. Hence, in part, early treatment reduces its mortality. However, the 5-year survival rate of HCC is still very low [6].

Multi-step processes including genetic and epigenetic alterations are thought to play a cumulative role in the progression of HCC [7]. Most of the abnormally expressed genes play a key role in the process of the malignant transformation of liver cells, such as the regulation of the cell cycle, cell growth, apoptosis, cell migration and diffusion [8]. The Wnt/β-catenin signalling pathway, generally activated by genetic and epigenetic alterations, has been linked to several types of tumours, including HCC [9]. Common epigenetic changes include DNA hypermethylation in the promoter region of WIF-1 [10]. The screening and identification of molecular targets involved in hepatic cell malignant transformation are very important, and these may become potential clinical therapeutic targets in HCC patients [11].

Integrator complex subunit 6 (INTS6), which was previously known as the gene encoding deleted in cancer cells 1 (DICE1) (OMIM 604331), was identified to localize with the microsatellite marker D13S284 in 13q14.3, a region frequently affected by allelic deletion in many solid tumours, such as prostate carcinoma, cervical carcinoma and oesophageal squamous cell carcinoma [12–14]. Some studies have identified the promoter of the tumour suppressor gene INTS6, which is down-regulated in prostate cancer, and have revealed that the INTS6 promoter is hypermethylated in prostate cancer cell lines [15].

Although INTS6 is known to play a key role in many solid tumours, including in HCC [16], the relationship between INTS6 expression and the clinicopathological characteristics of HCC and its molecular mechanisms are poorly unknown. The current study detected the expression of INTS6 in HCC using quantitative reverse transcriptase polymerase chain reaction (qRT-PCR), Western blotting, and immunohistochemistry analyses. After these experiments, we wanted to determine the relationship between INTS6 expression levels and the clinicopathological features of HCC and one of its pathways.

## Methods

### Patients and HCC tissue samples.

All of the clinical samples (including HCC tissues and adjacent normal liver tissues) were obtained from the Third Affiliated Hospital, Sun Yat-sen University (Guangzhou, China). All of the patients gave informed consent. This project was approved by the Clinical Research Ethics Committee of the Third Affiliated Hospital, Sun Yat-sen University.

Hepatocellular carcinoma tissues and their matched adjacent normal tissues (not less than 2 cm away from the tumour) were obtained from 3 patients and were used for the discovery of specific mRNA changes from the microarrays. A total of 50 HCC tumour tissues and matched adjacent normal liver tissues were obtained from patients, and 70 FFPE samples with pathologist-diagnosed HCC were obtained from the Third Affiliated Hospital of Sun Yat-sen University between the years 2008 and 2012. The liver and tumour tissues were immediately frozen in liquid nitrogen after surgery and stored at −80 °C until the extraction of total RNA.

We used the TNM classification of the 6th edition American Joint Committee on Cancer (AJCC) to classify the tumour stage. The patients included both men and women with ages ranging from 29 to 71 (mean age: 48.2 years). None of the patients who participated in this study received any pre-operative treatments, including TACE or radiofrequency ablation.

### Cell lines and culture condition

The HCC cell lines MHCC97L (catalogue number CC0109), Huh7 (catalogue number TCHu182), Hep3B (catalogue number TCHu106) and HepG2 (catalogue number TCHu 72) and a normal human hepatocyte (HH) (catalogue number GNHu 6) cell line were obtained from the Cell Bank of the Chinese Academy of Sciences, and all the cell lines were grown in DMEM (Gibco, Invitrogen, USA) supplemented with 10% foetal bovine serum (FBS) (Gibco, Invitrogen, USA), penicillin (100 units/ml) and streptomycin (100 units/ml) in 5% CO2 at 37 °C in a humidified incubator.

### HCC mRNA microarray analysis

The Arraystar Human lncRNA Array v2.0 was used to profile both lncRNAs and messenger RNAs (mRNAs) in the human genome of 3 pairs of human HCC and the matched normal tissues. Sample labelling and array hybridization were performed according to the Agilent One-Color Microarray-Based Gene Expression Analysis protocol (Agilent Technologies) with minor modifications [15] (the data have been deposited in the GEO: GSE64633).

### Plasmid DNA construction and transfection

The MSCV-based bicistronic retroviral vector MIEG3 was used to express INTS6 as described in our previous study [16]. All plasmid DNAs were verified by DNA sequencing. For plasmid transfection, HCC cell lines were grown in 6-well plates; the next day, cells were transfected with Lipofectamine 2000 (Invitrogen) according to the manufacturer’s recommendations. siRNA oligonucleotides targeting INTS6 or the negative control were transfected into the HCC cell lines using Lipofectamine RNAiMAX (Invitrogen, USA), according to the manufacturer’s protocol. Target gene expression levels were measured 72 h post-transfection. RNA and protein were acquired 72 h after transfection.

### Western blot analysis

Total frozen HCC and adjacent normal liver tissue proteins and HCC cell proteins were extracted using RIPA lysis buffer (KeyGen, China) according to the manufacturer’s’ instructions. For Western blotting, primary polyclonal antibodies against INTS6 (rabbit anti-INTS6, Abcam, 1:2000 dilution), WIF-1 (rabbit anti-WIF-1, Abcam, 1:2000 dilution), and β-catenin (rabbit anti-Beta Catenin, Abcam, 1:5000 dilution) were used. An actin antibody served as the internal control. The online software ImageJ was used to quantify the density of the bands.

### Quantitative real-time PCR analysis

All the RNA samples were reverse transcribed to synthesize cDNA using the PrimeScript® RT reagent kit with a gDNA Eraser (Takara, China).

QRT-PCR was employed to determine the relative expression levels of the target genes using a LightCycler 480 SYBR Green I Master (Roche).

The qRT-PCR reactions were performed in triplicate using the LC480 Real-Time PCR Detection System (Roche).

The primers for qRT-PCR were as follows: INTS6 forward 5′-AGCTGCCAGTTCTTGGAATG-3′ and reverse 5′-AGGCCAGACAGCTCTGATGT-3′; GAPDH, forward 5′-GTCCACCACCCTGTTGCTGTA-3′ and reverse 5′-CTTCAACAGCGACACCCACTC-3′. Additionally, we detected the expression levels of the downstream target genes ZEB1 and MMP13 from the Wnt/β-catenin pathway. The primers for qRT-PCR were as follows: ZEB1 forward 5′ -TGCACTGAGTGTGGAAAAGC-3′ and reverse 5′- TGGTGATGCTGAAAGAGACG-3′; MMP13 forward 5′ - ACTGAGAGGCTCCGAGAAATG-3′ and reverse 5′- GAACCCCGCATCTTGGCTT-3′.

The cycle at which the reaction crossed an arbitrarily placed threshold (Ct) was determined for each gene. The mRNA expression level of each gene was calculated using the △Ct method: △Ct = CtmRNA – CtGAPDH.

### Immunohistochemistry

A total of 70 HCC tissue samples were collected by the Department of Pathology, and all the samples were fixed in formalin and embedded in paraffin.

The HE-stained sections of each specimen from a single random block were measured by a senior pathologist.

Both percent positivity and staining intensity of tumour cells were viewed according to a double blinded method. Immunohistochemistry was performed based on a previously described method [17]. The percent positivity was scored as “0” (<5%, negative), “1” (5–25%, sporadic), “2” (25–50%, focal), or “3” (>50%, diffuse). The staining intensity was scored as “0” (no staining), “1” (weakly stained), “2” (moderately stained), or “3” (strongly stained). The final INTS6 expression score was calculated using the value of the percent positivity cell score × the staining intensity score [18], which ranged from 0 to 9. The INTS6 expression level was defined as follows: “−” (score 0–1), “+” (score 2–4), “++” (score 5–6), and “+++” (score > 6). According to previous research, a low expression level of INTS6 was defined as a total score ≤ 4, and a high expression level of INTS6 was defined as a total score > 4.

### Statistical analysis

The statistical analyses were performed with SPSS 14.0 software. A *p*-value of <0.05 was considered significant. The non-parametric statistical test was used to analyse the differences in the protein and mRNA expression levels of INTS6 in HCC tissue and cell lines. The χ^2^ test was used to analyse the relationship between the expression levels of INTS6 and the clinicopathological characteristics of HCC patients. Survival curves were calculated using the Kaplan-Meier method and were compared using the log-rank test. Finally, univariate and multivariate Cox regression analyses were used to evaluate the survival rates of HCC patients.

## Results

### Expression of INTS6 mRNA in HCC patients determined by microarray analysis

**Fig. 1:**
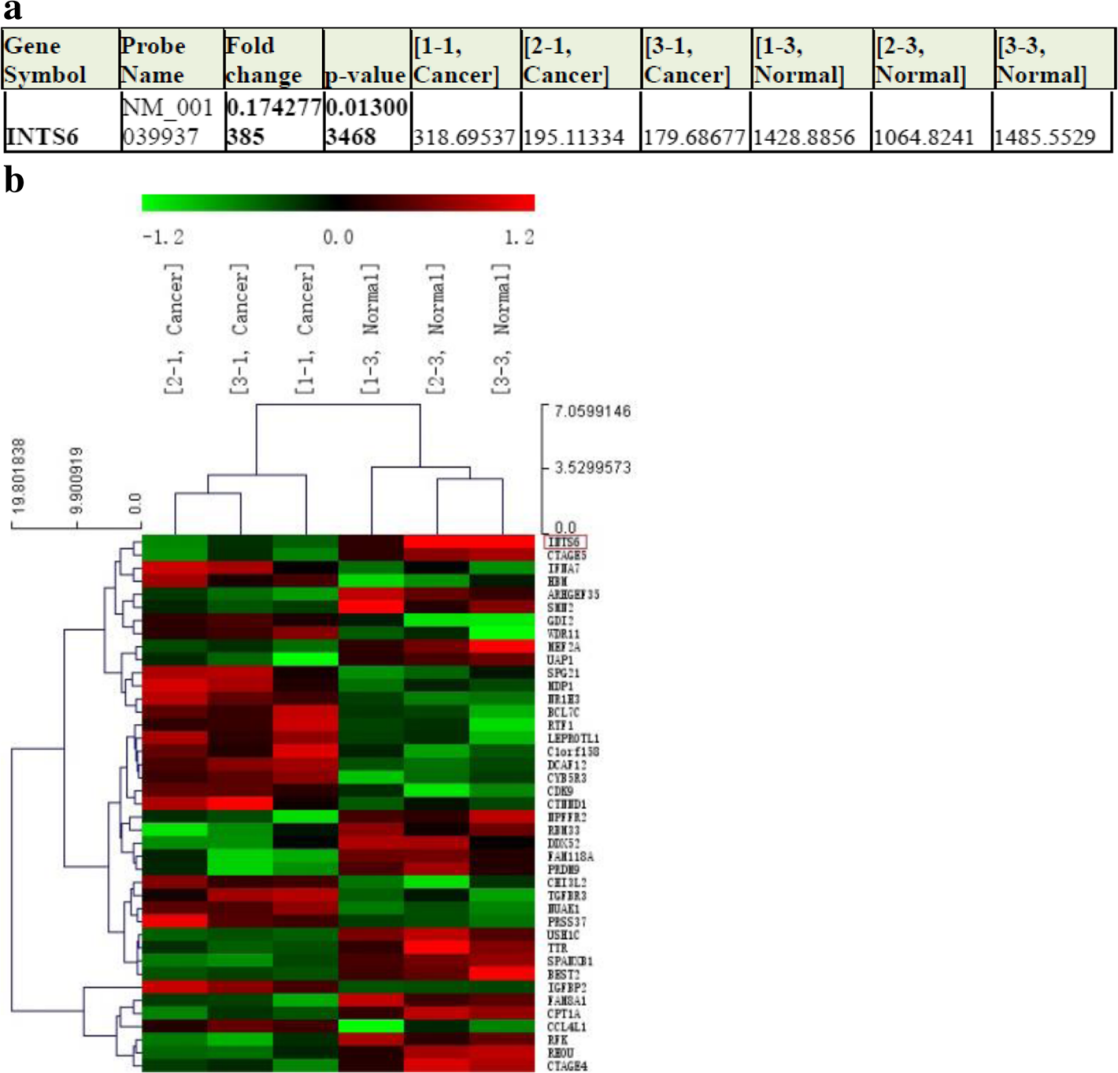
**a**. INTS6 microarray data from HCC tissues vs. normal adjacent tissues (3 pairs, *p* < 0.01). **b**. Heat map from the microarray of HCC tissues vs. normal adjacent tissues

To discover the dysregulated genes in HCC tissue, we collected 3 HCC tissues and their matched adjacent normal tissues and used microarray analysis. According to the microarray data analysis (2.0-fold up- or down-regulated and *p*<0.05) (the data have been deposited in the GEO: GSE64633), the expression levels of INTS6 were dramatically down-regulated in HCC vs. matched normal liver tissues (*p* = 0.015) (Fig. 1a, b). Detailed information and the analysis file for the differential mRNA expression are summarized in the supplementary material (Additional files 1).

### INTS6 mRNA and protein expression was down-regulated in HCC compared to the corresponding adjacent liver tissues

To further explore the array data, we collected a larger cohort of human HCC and paired normal liver tissues and detected the expression levels of INTS6. We found that the INTS6 expression was down-regulated in 68.0% (34/50) of the HCC tissues, compared to the expression in the normal liver tissues.

**Fig. 2:**
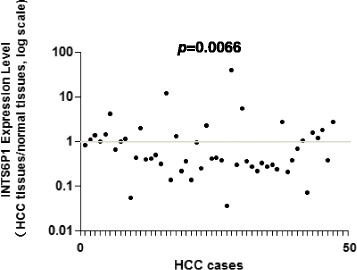
Relative expression level of INTS6 mRNA in 50 paired HCC tissues and adjacent normal tissues by qRT-PCR (*p* = 0.0066)

**Fig. 3:**
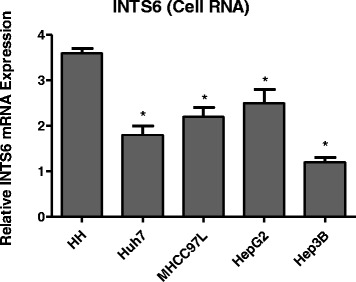
qRT-PCR analysis of INTS6 expression levels in different liver cell lines. GAPDH was used as an endogenous control for normalizing experimental data. *p<0.05

**Fig. 4:**
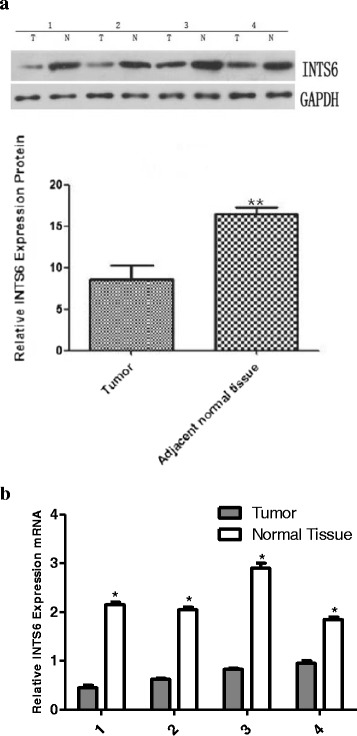
**a**. Expression of INTS6 protein in HCC and adjacent normal tissue samples using Western blotting (T = tumour, N = normal tissue). *p<0.05, **p<0.01. **b**. Expression of INTS6 mRNA in the corresponding HCC and adjacent normal tissue samples (T = tumour, N = normal tissue). *p<0.05, **p<0.01

From the analysis of the 50 paired HCC samples, we identified a remarkable difference in the expression levels of INTS6 between the HCC and adjacent liver tissues (*p* = 0.0066, Fig. 2). What is more, the expression of INTS6 was down-regulated in HCC cell lines (Huh7, MHCC97L, HepG2 and Hep3B) when compared to normal human hepatocytes (HH) (*p*<0.05, Fig. 3). Then, the INTS6 protein levels in the same HCC samples used for qRT-PCR were examined by using Western blotting. From our results, both the INTS6 protein and mRNA expression levels were reduced in the HCC tissues compared to the corresponding adjacent normal tissues (Fig. 4a, b).

### INTS6 expression was correlated with the clinicopathological features of HCC

**Fig. 5:**
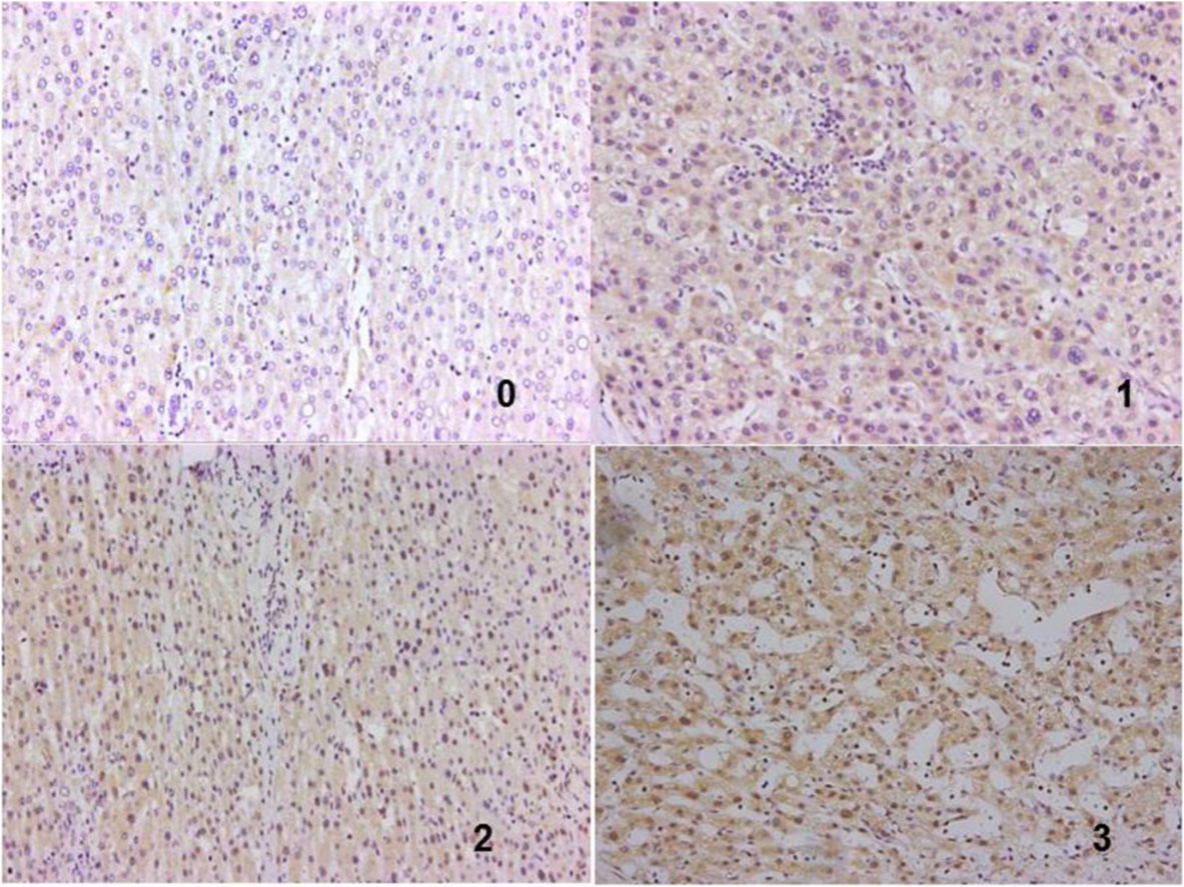
Immunohistochemical staining of INTS6 in HCC. INTS6 protein expression localized mainly to the nuclei in tumour cells. Different INTS6 staining intensities [negative: 0, weak: 1, moderate: 2, strong: 3] are indicated in the micrographs

Immunohistochemistry was performed on 70 archived paraffin-embedded HCC samples. The results revealed that INTS6 expression localized primarily to the nuclei of tumour cells (Fig. 5). Low INTS6 expression was present in 44 (62.9%) of 70 HCC cases. The correlation between INTS6 expression and the clinicopathological features of HCC was analysed using the chi-square test (Table 1). The expression level of INTS6 was significantly associated with the serum alpha-fetoprotein level (AFP, *p* = 0.004), pathology grade (*p* = 0.005), and tumour recurrence (*p* = 0.040). Moreover, from our qRT-PCR analysis of HCC tissues and adjacent normal liver tissues (Table 2), the expression level of INTS6 was remarkably associated with the serum alpha-fetoprotein levels (*p* = 0.004) and pathology grade (*p* = 0.006). Therefore, HCC patients with low INTS6 expression had a higher tendency to have high AFP levels, a poor pathology grade, and tumour recurrence. However, neither immunohistochemistry nor qRT-PCR analyses of INTS6 expression had statistically significant associations with age, gender, HBsAg positivity, tumour size, or cirrhosis.

**Table 1:** Correlation between expression level of INTS6 and clinicopathlogical features of HCC in Immunohistochemistry

clinicopathological features	INTS6 expression		Total	*P*-value
	low	high		
All cases	44	26	70	
Age				0.808
≥ 50	17	13	30	
<50	27	13	40	
Gender				0.104
Male	40	23	63	
Female	4	3	7	
HBsAg				0.096
Positive	37	22	59	
Negative	7	4	11	
Serum AFP(ng/ml)				0.004*
≤ 20	9	16	25	
>20	35	10	45	
Tumor size(cm)				0.387
≥ 5	18	6	24	
<5	26	20	46	
Pathology grade				0.005*
I(well)	4	15	19	
II(moderate)	37	11	48	
III(poor)	3	0	3	
Cirrhosis				0.197
Yes	38	17	55	
No	6	9	15	
Recurrence				0.040*
Yes	35	10	45	
No	9	16	25	
Vascular invasion				0.378
Yes	24	3	27	
No	20	23	43	

*p<0.05

**Table 2:** Correlation between expression level of INTS6 and clinicopathlogical features of HCC in qRT-PCR

clinicopathological features	INTS6 expression		Total	*P*-value
	low	high		
All cases	34	16	50	
Age				0.154
≥ 50	14	4	18	
<50	28	12	40	
Gender				0.204
Male	28	15	43	
Female	6	1	7	
HBsAg				0.495
Positive	31	14	45	
Negative	3	2	5	
Serum AFP(ng/ml)				0.004*
≤ 20	10	11	21	
>20	24	5	45	
Tumor size(cm)				0.314
≥ 5	19	3	22	
<5	15	13	28	
Pathology grade				0.006*
I(well)	2	5	7	
II(moderate)	28	11	39	
III(poor)	4	0	4	
Cirrhosis				0.233
Yes	25	9	34	
No	9	7	16	
Vascular invasion				0.169
Yes	13	2	15	
No	21	14	35	

*p<0.05

### Low expression of INTS6 was correlated with poor prognosis in HCC patients

**Fig. 6:**
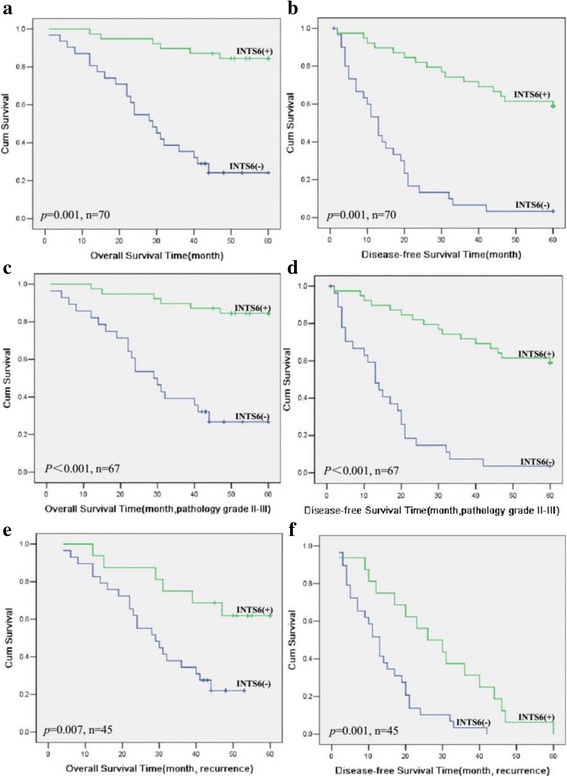
Kaplan-Meier analysis of overall and disease-free survival time in HCC patients (log-rank test, *p* = 0.001 and *p* = 0.001) (**a-b**). Kaplan-Meier analysis of overall survival and disease-free survival rates in subclassified HCC patients Stratified survival analysis of overall and disease-free survival rates according to poor pathology grades II–III (**c–d**) and tumour recurrence (**e–f**)

Survival curves were plotted using the Kaplan-Meier method and were compared using the log-rank test for 70 archived paraffin-embedded HCC patient samples. Patients with low INTS6 expression had shorter overall and disease-free survival time than patients with high INTS6 expression (*p* = 0.001 and *p* = 0.001, Fig. 6a-b). Stratified survival analysis explored the prognostic value of INTS6 according to poor pathology grade and tumour recurrence. Patients with low INTS6 expression had shorter survival time with poor pathology grades II–III and tumour recurrence (*p*<0.001 and *p* = 0.007, Fig. 6c, e). In addition, they also had shorter disease-free survival time with poor pathology grades II–III and tumour recurrence (*p*<0.001 and *p =* 0.001, Fig. 6d, f) than patients with high INTS6 expression.

### INTS6 was an independent predictor of overall and disease-free survival rates in HCC patients

Univariate and multivariate Cox analyses explored the impacts of the expression of INTS6 and other clinicopathological parameters on the overall and disease-free survival rates in 70 archived paraffin-embedded HCC patient samples. Univariate Cox regression analyses revealed that the expression of INTS6 (*p* = 0.001), pathology grade (*p* = 0.005), tumour size (*p* = 0.035), vascular invasion (*p* = 0.001) and recurrence (*p* = 0.002) correlated with overall survival (Table 3). In addition, multivariate Cox regression analysis also indicated that the expression of INTS6 (*p* = 0.040), recurrence (*p* = 0.041) and vascular invasion (*p* = 0.024) were independent predictors of overall survival in HCC patients (Table 2). Furthermore, the expression of INTS6 (*p* = 0.001), tumour size (*p* = 0.004), pathology grade (*p* = 0.009) and vascular invasion (*p* = 0.001) were correlated with disease-free survival (Table 3). In addition, multivariate Cox regression analysis also indicated that the expression of INTS6 (*p* = 0.025) and vascular invasion (*p* = 0.001) were independent predictors of disease-free survival in HCC patients (Table 4).

**Table 3:** Univariate analysis and multivariate analysis overall survival time

Variables	Univariate analysis				Multivariate analysis			
	*P* value	Exp(B)	95.0% CI		*P* value	Exp(B)	95.0% CI	
			Lower	Upper			Lower	Upper
Gender	0.911	0.934	0.282	3.094				
Age	0.240	0.980	0.948	1.013				
Pathology grade	0.005*	0.331	0.152	0.720				
HBsAg	0.626	1.300	0.452	3.736				
AFP	0.091	2.023	0.894	4.577				
Tumor size	0.035*	2.196	1.055	4.568				
Cirrhosis	0.079	2.919	0.883	9.654				
Recurrence	0.002*	23.148	3.136	170.849	0.041*	9.110	1.054	75.895
Vascular invasion	<0.001*	7.685	3.340	17.682	0.024*	2.847	1.151	7.046
INTS6	<0.001*	0.125	0.038	0.416	0.040*	0.221	0.047	1.304

*p<0.05

**Table 4:** Univariate analysis and multivariate analysis disease-free survival time

Variables	Univariate analysis				Multivariate analysis			
	*P* value	Exp(B)	95.0% CI		*P* value	Exp(B)	95.0% CI	
			Lower	Upper			Lower	Upper
Gender	0.193	1.778	0.748	4.227				
Age	0.443	0.990	0.964	1.016				
Pathology grade	0.009*	0.409	0.209	0.803				
HBsAg	0.460	1.383	0.585	3.269				
AFP	0.081	1.741	0.934	3.246				
Tumor size	0.004*	2.398	1.317	4.367				
Cirrhosis	0.077	2.076	0.925	4.660				
Vascular invasion	<0.001*	6.724	3.542	12.764	<0.001*	5.013	2.415	10.408
INTS6	<0.001*	0.266	0.131	0.542	0.025*	0.425	0.201	0.896

*p<0.05

### INTS6 inhibited the Wnt/β-catenin signalling pathway by decreasing the expression levels of WIF-1

**Fig. 7:**
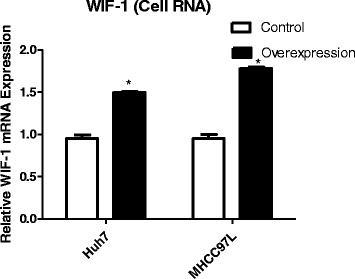
Relative expression levels of WIF-1 mRNA in the HCC cell lines Huh7 and MHCC97L by qRT-PCR. *p<0.05

**Fig. 8:**
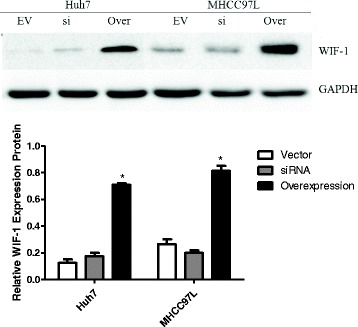
Expression of WIF-1 protein in the HCC cell lines Hun7 and MHCC97L using Western blotting (EV = Vector, si = siRNA, Over = Overexpression). *p<0.05

**Fig. 9:**
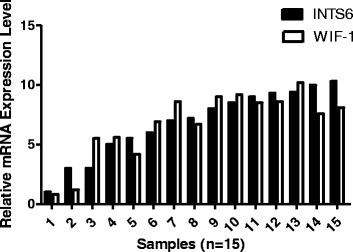
Relative expression levels of WIF-1 and INTS6 mRNA in 15 HCC tumour tissues by qRT-PCR. The relative mRNA level was normalized to GAPDH

**Fig. 10:**
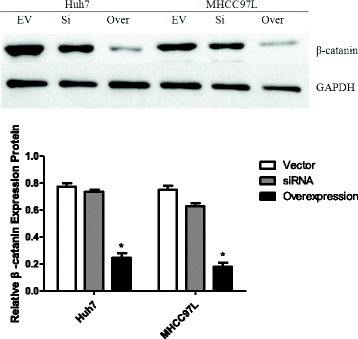
Expression of *β*-catenin protein in the HCC cell lines Hun7 and MHCC97L using Western blotting (EV = Vector, si = siRNA, Over = Overexpression). *p<0.05

**Fig. 11:**
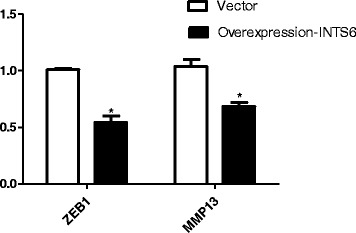
Expression of downstream target genes MMP13 and ZEB1 measured by qRT-PCR. *p<0.05

qRT-PCR analysis showed that WIF-1 mRNA levels in the HCC cell lines were significantly increased by INTS6 overexpression compared with those in the control cells (Fig. 7). Consistent with the qRT-PCR results, the protein expression levels of WIF-1 in Huh7 and MHCC97L cells were dramatically increased after INTS6 overexpression (Fig. 8). What is more, the protein levels of WIF-1 were slightly changed by the knockdown of INTS6 by siRNA. Furthermore, the inverse correlation between INTS6 and WIF-1 expression was also detected in 15 HCC clinical tissues by using qRT-PCR (Fig. 9). Then, the concentrations of β-catenin in HCC in Huh7 and MHCC97L cells were measured, and decreased β-catenin expression was observed after INTS6 overexpression by Western blot analysis (Fig. 10). Additionally, we found that the downstream target genes just like ZEB1 and MMP13, which play an important role in tumour regulation, were decreased in the INTS6 overexpressing cells (Fig. 11). Based on our results, it was suggested that INTS6 increased WIF-1 expression and then inhibited the Wnt/β-catenin signalling pathway.

## Discussion

Currently, many epigenetic changes are found in HCC and play a crucial role in aetiology. In addition, mRNA microarray analyses revealed that the gene expression changes in HCC were variable, with some types of HCC being defined based on its mRNA levels [19, 20]. From our mRNA microarray results, the expression level of INTS6 has been shown to be dramatically down-regulated in HCC vs. normal liver tissues. Therefore, we report that INTS6 is a potential and significant potent tumour suppressor in HCC. Several studies have suggested that INTS6 plays an important role as a tumour suppressor in some human cancers. In mechanistic studies, INTS6 tends to induce the Gap 1 (G1) arrest, thus explaining its tumour suppressor role in prostate cancer [18]. Moreover, another study of INTS6 in prostate cancer shows that lower expression of INTS6 can cause hypermethylation of the promoter region CpG [9]. Moreover, INTS6 functions are involved in cell cycle regulation and the cell-cell communication pathway, similar to its regulatory role in the Wnt signalling pathway [18, 21]. However, the relationship between INTS6 expression and the clinicopathological characteristics of HCC and its mechanism are still largely unknown.

Here, we are the first to report that the down-regulation of INTS6 strongly correlates with high AFP levels, poor pathology grades, and tumour recurrence. Some studies show that serum AFP levels have considerable predictive value for HCC malignancy and prognosis [22]. HCC patients with AFP levels ≤20 ng/ml may benefit the most from hepatectomy as a primary treatment, but patients with AFP levels >20 ng/ml need comprehensive therapy, surgical resection, and close follow-up examinations. Cirrhosis is commonly considered one of the most important factors associated with HCC prognosis [23]. However, in our study, we found that INTS6 expression is not associated with cirrhosis. Therefore, we propose that the expression level of INTS6 was largely due to the tumour itself rather than cirrhosis and HBsAg-positivity.

It has been reported that some clinicopathology features, including tumour size, nodule number, macro/μ invasion and pre-operative AFP, are considered as survival indices that affect the prognosis of HCC patients. Recently, a correlation between obesity, lifestyle factors (smoking, drinking status and exercise) and HCC prognosis was emphasized. However, there is not enough evidence to prove the relationships between clinical features and HCC prognosis. Therefore, if we want to gain a thorough understanding of how those factors affect the prognosis and provide the basis for personalized therapy for our patients, molecular studies may be the key to open the door. In our study, we found that the correlation between a higher level of AFP (AFP ≥ 20 ng), lower pathology grade (poor) and tumour recurrence and a lower expression level of INTS6 may be associated with poor prognosis in HCC patients. Moreover, survival curves indicate that patients with low INTS6 expression have shorter overall and disease-free survival rates. Our results strengthen the hypothesis that low INTS6 expression is associated with a poor prognosis in HCC patients.

Cox regression analysis also showed that INTS6 and vascular invasion are the independent predictors of overall survival and disease-free survival. The reasons why AFP, tumour size, pathology grade are not independent predictors for HCC may be due to the effects of multiple collinearity and the small sample size. It suggested that INTS6 may be used as a new prognostic marker to identify HCC patients at a high risk of poor prognosis. The current data show that INTS6 could be used as a potential and independent predictor of prognosis in HCC.

Mechanistically, the INTS6/WIF-1 regulatory model investigated in this study provides a new perspective on WIF regulation. In fact, many studies have reported that WIF-1 down-regulation is involved in tumours, including in HCC [24–26], and could trigger the Wnt/β-catenin signal pathway [27]. WIF-1 is one of the endogenous antagonists that inhibits the Wnt pathway by directly binding to Wnt proteins in the extracellular space [28]. Down-regulation of the expression level of WIF-1 due to its hypermethylated promoter has been reported in bladder cancer, melanoma, lung cancer, and HCC [29]. Moreover, in Hu’s study [30], it was shown that Wnt antagonists WIF1-Fc and SFRP1-Fc inhibit Wnt signalling and exert antitumour activity by inducing apoptosis in tumour cells, which indicates that Wnt antagonists would be a promising molecular treatment for HCC. All the above studies indicate that WIF-1 plays an important role in the Wnt/β-catenin signalling pathway, especially in HCC. In our previous study [16], we learned that INTS6 inhibits HCC cell growth and migration and promotes apoptosis. We also found that INTS6 functions as a competitive endogenous RNA (ceRNA) to up-regulate its tumour suppressor pseudogene INTS6P1 to inhibit HCC. In this study, it was hypothesized that INTS6 overexpression inhibited the WNT signal pathway by increasing WIF-1 expression. As expected, there was a significantly decrease in the expression level of β-catenin in the HCC cell lines, and the downstream target genes ZEB1 and MMP13 were decreased after INTS6 overexpression.

In conclusion, this is the first report to demonstrate the clinical significance of INTS6 expression and its mechanism in HCC. This research is limited by its sample size and the inclusion of only patients with resectable tumours. Larger prospective studies examining INTS6 are necessary to further validate the usefulness of this biomarker. INTS6 overexpression may prevent invasive progression and metastatic relapse, which would improve the prognosis and quality of life for HCC patients after hepatic resection.

## Conclusions

The results of our study show that down-regulated INTS6 expression was associated with a poor prognosis in HCC patients. This newly identified INTS6/WIF-1 axis indicates the molecular mechanism of HCC and may represent a therapeutic target in HCC patients.

## Additional file

Additional file 1: Microarray data files of the differential mRNA expression in HCC. (XLS 2230 kb)

## Abbreviations

AJCC: American Joint Committee on Cancer
DICE1: Deleted in cancer cells 1
FFPE: Formalin-fixed paraffin-embedded
HCC: Hepatocellular carcinoma
IHC: Immunohistochemistry
INTS6: Integrator complex subunit 6
MMP13: Matrix Metallopeptidase 13
qRT-PCR: Quantitative reverse transcriptase polymerase chain reaction
ZEB1: Zinc finger E-box-binding homeobox 1
